# Distinct Effects of the Apolipoprotein E ε4 Genotype on Associations Between Delayed Recall Performance and Resting-State Electroencephalography Theta Power in Elderly People Without Dementia

**DOI:** 10.3389/fnagi.2022.830149

**Published:** 2022-05-26

**Authors:** Jing Wang, Tingting Sun, Ying Zhang, Xin Yu, Huali Wang

**Affiliations:** ^1^Peking University Institute of Mental Health (Sixth Hospital), Beijing, China; ^2^National Clinical Research Center for Mental Disorders, NHC Key Laboratory of Mental Health, Peking University, Beijing, China; ^3^Beijing Municipal Key Laboratory for Translational Research on Diagnosis and Treatment of Dementia, Beijing, China; ^4^Department of Psychiatry, The Third Affiliated Hospital, Sun Yat-sen University, Guangzhou, China

**Keywords:** elderly people, delayed recall (memory), resting-state EEG, theta power, apolipoprotein E

## Abstract

**Background:**

Abnormal electroencephalography (EEG) activity has been demonstrated in mild cognitive impairment (MCI), and theta rhythm might be inversely related to memory. The apolipoprotein E (ApoE) epsilon 4 (ε4) allele, as a genetic vulnerability factor for pathologic and normal age-related cognitive decline, may influence different patterns of cognitive dysfunction. Therefore, the present study primarily aimed to verify the role of resting theta rhythm in delayed recall deficits, and further explore the effects of the ApoE genotype on the associations between the resting theta power and delayed recall performance in the elderly individuals without dementia.

**Methods:**

A total of 47 individuals without dementia, including 23 MCI and 24 healthy subjects (HCs), participated in the study. All subjects were administered the Hopkins Verbal Learning Test–Revised (HVLT-R) to measure delayed recall performance. Power spectra based on resting-state EEG data were used to examine brain oscillations. Linear regression was used to examine the relationships between EEG power and delayed recall performance in each subgroup.

**Results:**

The increased theta power in the bilateral central and temporal areas (*P*_*s*_ = 0.02–0.044, uncorrected) was found in the patients with MCI, and were negatively correlated with delayed recall performance (r_s_ = −0.358 to −0.306, *P*_s_ = 0.014–0.036, FDR corrected) in the elderly individuals without dementia. The worse delayed recall performance was associated with higher theta power in the left central and temporal areas, especially in ApoE ε4 non-carriers and not in carriers (*r*_s_ = −0.404 to −0.369, *P*_s_ = 0.02–0.035, uncorrected).

**Conclusion:**

Our study suggests that theta disturbances might contribute to delayed recall memory decline. The ApoE genotype may have distinct effects on EEG-based neural correlates of episodic memory performance.

## Introduction

Individuals with mild cognitive impairment (MCI) have a clinically observable cognitive deficit and are likely to progress to dementia (Gauthier et al., 2006; Reisberg and Gauthier, 2008; Melrose et al., 2018). Deficits in delayed recall of episodic memory have been considered a potential predictor of the conversion from MCI to Alzheimer’s disease (AD) (Gainotti et al., 2014).

A growing literature has demonstrated an increase in theta oscillation accompanied by a decreased alpha and beta power as the most stable pattern of oscillation activity in MCI patients (Grunwald et al., 2002; Prichep et al., 2006; Lopez et al., 2014). Previous studies suggested that the resting and task-evoked electroencephalography (EEG) theta rhythm might be inversely related to memory (Claus et al., 1998; Musaeus et al., 2018; Goodman et al., 2019). Recently, Borhani et al. (2021) reported that faster working memory retrieval was significantly correlated with increased resting-state delta and theta band powers over the left parietal sites in healthy older adults. A recent simultaneous EEG-functional magnetic resonance imaging (fMRI) study observed that individuals with MCI exhibited chaotic event-related potentials during the word retrieval task while preserving the spatial activity pattern (Shu et al., 2021). However, it remains obscure that whether the resting theta power is linked to the delayed recall memory in the elderly people without dementia.

The ε4 allele of the apolipoprotein E gene (ApoE) is a well-established genetic vulnerability factor for pathologic and normal age-related cognitive decline (Todd et al., 2018; Slot et al., 2019; Williams et al., 2019; Yin et al., 2019; Emrani et al., 2020; Montagne et al., 2020). Although negative effects associated with this gene on cognition have been found in ApoE ε4 carriers, increasing researches suggest that ε4 allele carriers and non-carriers may suffer from different patterns of cognitive dysfunction (Fan et al., 2019) and brain activities. For example, Wolk et al. (2010) found ε4 carriers in AD exhibited decreased verbal memory, while non-carriers exhibited poor working memory, executive function, and verbal ability. A number of studies have suggested that carriers exhibit greater MTL atrophy (Pievani et al., 2009; Wolk and Dickerson, 2010; Prieto Del Val et al., 2018; Emrani et al., 2020; Kim et al., 2021), dysfunctional EEG oscillations (Michels et al., 2017; Prieto Del Val et al., 2018), and more complex brain activity (Gutiérrez-de Pablo et al., 2020). Recent research has demonstrated ApoE ε4-mediated modulation of intrinsic functional brain network architecture in cognitively normal individuals and patients with AD (Aad et al., 2015). Nevertheless, how the ApoE genotype affects the EEG correlates of delayed recall is still poorly understood.

Therefore, the purpose of the present study was to verify the role of resting theta rhythm in delayed recall deficits, and further explore the modulating effects of the ApoE genotype on the associations between the resting theta power and delayed recall performance in the elderly individuals without dementia. We hypothesized that the theta rhythm might be linked with delayed recall performance and these relationships might be affected by the ApoE genotype status.

## Materials and Methods

### Participants

Forty-seven elderly participants without dementia, including 23 subjects with amnestic MCI and 24 healthy subject (HCs), were recruited from the Dementia Care & Research Center of Peking University Institute of Mental Health from May 2018 to November 2019. The inclusion criteria were as follows: (a) right-handedness; (b) age ≥ 55 years; (c) more than 5 years of schooling education; (d) a Hamilton Depression Scale (HAMD) score of < 12; (e) preserved general cognitive function [Mini-Mental State Examination (MMSE) score of > 24], (f) intact activities of daily living (ADL score of ≤ 26), (g) a Clinical Dementia Rating (CDR) score ≤ 0.5, and (g) failure to meet the diagnosis of dementia (Hughes et al., 2019). All participants with MCI met the Petersen’s amnestic MCI criteria. All healthy participants underwent neuropsychological assessments and CDR to exclude cognitive impairment.

The exclusion criteria included psychotic episodes listed in the Diagnostic and Statistical Manual of Mental Disorders 4th edition (DSM-IV), history of stroke, subdural hematoma, tumor, other intracranial space-occupying diseases or cerebrovascular disorders, and presence of significant risk factors for cerebrovascular disorders, current or previous neuropsychiatric diseases such as Parkinson’s disease or epilepsy; and presence of a physical illness that could affect cognition.

The present study was approved by the ethics committee of Peking University Institute of Mental Health (Sixth Hospital), Beijing, China. All participants were fully informed of the study protocol and provided written informed consent.

### Neuropsychological Tests

All participants underwent a neuropsychological assessment. For the purpose of this study, we included the MMSE, the Montreal Cognitive Assessment (MoCA), and Hopkins Verbal Learning Test–Revised (HVLT-R). Considering that MMSE and MoCA can be affected by different linguistic, educational, cultural and socioeconomic backgrounds, these two measures were only used as the screening tools to evaluated the individuals’ suitability for inclusion/exclusion. The delayed recall scores on the HVLT (HVLT-DR) measured delayed recall performance.

### Apolipoprotein E Genotyping

Apolipoprotein E genotyping was performed as previously described (Wang X. et al., 2015). The participants were classified as either ApoE ε4 positive/carriers (genotype of ε3/ε4 or ε4/ε4) or ApoE ε4 negative/non-carriers (genotype of ε3/ε3 or ε2/ε3). The subjects with two copies of the ApoE ε2 allele were excluded due to its potential protective effect (Suri et al., 2013).

### Electroencephalography Data Acquisition and Preprocessing

Electroencephalography signals were collected with a 64-channel EEG system (bandpass: 0.01–100 Hz; Brain Products GmbH, Munich, Germany). Electrode impedance was kept below 20 kΩ. All subjects were comfortably seated in a sound-attenuating room and kindly asked to stay relaxed and awake with their eyes closed and not to move or talk; then, 5 min of EEG signals were continuously collected.

Signals were analyzed offline with the MATLAB R2014a (The Mathworks, Natick, MA, United States)-based EEGLAB toolbox.^1^ All recorded artifact-free EEG data were resampled to 500 Hz, rereferenced to an average of residual channels, bandpass filtered in the range of 1–45 Hz to avoid the interference of 50-Hz signals. The data were subsequently manually inspected for conspicuous baseline drift, eye movements, muscle or any other non-physiological artifact rejection, according to the visually identified waves. The artifact rejection threshold was set to 100 μV.

To detect EEG power in different regions, the brain was divided into eight brain regions: left frontal lobe (LF, electrodes: Fp1, F1, F3, F5, F7, AF3, AF7), right frontal lobe (RF, electrodes: Fp2, F2, F4, F6, F8, AF4, AF8), left central area (LC, electrodes: C1, C3, C5, FC1, FC3, FC5, CP1, CP3, CP5), right central area (RC, electrodes: C2, C4, C6, FC2, FC4, FC6, CP2, CP4, CP6), left temporal lobe (LT, electrodes: T7, TP7, TP9, FT7, FT9), right temporal lobe (RT, electrodes: T8, TP8, TP10, FT8, FT10), left parieto-occipital lobe (LPO, electrodes: P1, P3, P5, P7, PO3, PO7, O1), and right parieto-occipital lobe (RPO, electrodes: P2, P4, P6, P8, PO4, PO8, O2) (Bian et al., 2014).

### Electroencephalography Power Spectrum Analysis

The wavelet-based power spectrum analysis method was used to obtain the power of the resting-state EEG activities (Wang et al., 2017). The Morlet wavelet transform was employed Briefly, signals were divided into a series of 2-s epochs. For each epoch, the Morlet wavelet transform was applied with a wavelet central angle frequency of 6 (ω = 6) (Li et al., 2007). The power values in the frequency with a step of 0.5 Hz across all electrodes were computed for each epoch and then averaged across all the 2 s epochs. The average power values in following five frequency bands, were finally obtained: 1–4, 4–8, 8–13, 13–30, 30–45 Hz, corresponding to delta, theta, alpha, beta, and gamma bands, respectively (Mantini et al., 2007).

### Statistical Analysis

For the age in the demographic information, independent sample *t*-test was used to compare the differences between the MCI and NC group. The χ^2^ test was conducted to compare sex information. Considering the age effect on the cognition, the linear regressions with the age as the covariance were used to compare the differences on HVLT-DR and total cognitive scores. For the EEG power values, non-parametric Mann–Whitney tests were used to compare the difference.

The linear regression analyses were used to examine the relationships between EEG power values with the statistical significance and delayed recall performance.

The significance level of *P* < 0.05 was corrected by a false discovery rate (FDR) for the multiple comparisons.

## Results

There were no significant differences in sex, or education level between the MCI and HC groups. The MCI participants were elder (Cohen’s *d* = 5.75, *P* = 0.041), and performed worse than the controls on the MMSE (Cohen’s *d* = 2.76, *P* < 0.0001) and MoCA (Cohen’s *d* = 5.1, *P* < 0.0001). The HVLT-DR score was significantly lower in the MCI group than in the control group (Cohen’s *d* = 3.24, *P* = 0.012) (see Table 1).

**TABLE 1:** Demographics and cognitive performance of the elderly individuals without dementia grouped by MCI and NC.

	MCI (*N* = 23)	HC (*N* = 24)	F/*x*^2^	Cohen’s *d*	*P*
Age	72.21 ± 10.02	66.46 ± 7.75	4.435	5.75	0.041
Sex (male/female)	9/14	14/10	1.733	NA	0.188
Education	13.57 ± 2.61	13.21 ± 2.83	0.539	0.36	0.466
MMSE	26.57 ± 1.93	29.33 ± 1.13	29.524	2.76	< 0.0001***
MoCA	22.61 ± 2.13	27.71 ± 1.16	89.395	5.1	< 0.0001***
HVLT-DR	5.47 ± 3.73	8.71 ± 2.44	6.911	3.24	0.012*

*MMSE, Mini-Mental State Examination; MoCA, Montreal Cognitive Assessment; HVLT-DR, Hopkins Verbal Learning Test–Revised- Delayed Recall.*

*P < 0.05, ***P < 0.001, NA, not applicable.

**FIGURE 1:**
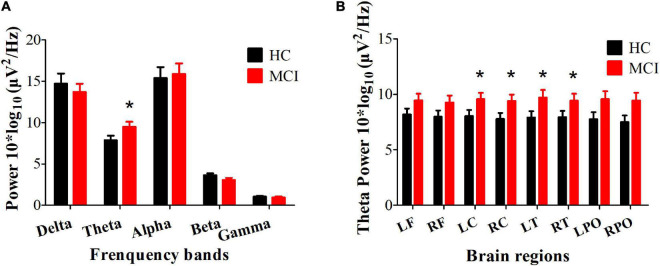
Differences in the resting EEG power spectra between HC and MCI group. **(A)** The average absolute EEG power in 5 frequency bands in the HC (black columns) and MCI (red columns) group. The *Y*-axis represents power values, and the *X*-axis represents the frequency bands. A marked increase in power in the theta frequency was observed in the MCI group. *P < 0.05, FDR uncorrected. All data are expressed as the means ± SEM. **(B)** Distribution of statistically significant theta power in the HC (black columns) and MCI (red columns) group. A marked increase in power in the theta frequency was observed in the bilateral central and temporal regions. *P < 0.05, FDR uncorrected. All data are expressed as the means ± SEM. LF, left frontal lobe; RF, right frontal lobe; LC, left central area; RC, right central area; LT, left temporal lobe; RT, right temporal lobe; LPO, left parieto-occipital lobe; RPO, right parieto-occipital lobe.

**FIGURE 2:**
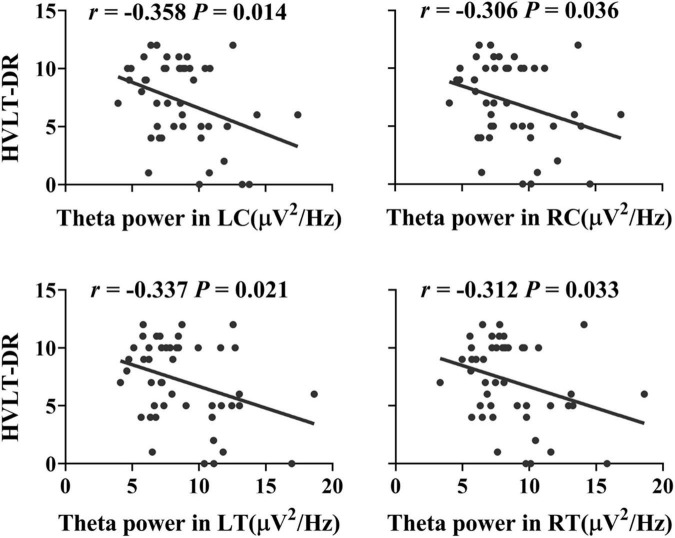
Associations between the delayed recall performance and the resting theta power in the bilateral central and temporal regions in the elderly individuals without dementia (*R*_LC_ = −0.358, *P*_LC_ = 0.014, *R*_RC_ = −0.306, *P*_RC_ = 0.036, *R*_LT_ = −0.337, *P*_LT_ = 0.021, *R*_RT_ = −0.312, *P*_RT_ = 0.033, *FDR* corrected). HVLT-DR, Hopkins Verbal Learning Test-Delayed Recall; LC, left central area; RC, right central area; LT, left temporal lobe; RT, right temporal lobe.

Compared with the HC group, the MCI group showed increased power in the theta band (Cohen’s *d* = 1.62, *P* = 0.034, uncorrected), mainly located in the central and temporal regions (Cohen’s *d*_LC_ = 1.53, *P*_LC_ = 0.023; Cohen’s *d*_RC_ = 1.61, *P*_RC_ = 0.025; Cohen’s *d*_LT_ = 1.80, *P*_LT_ = 0.044; Cohen’s *d*_RT_ = 1.49, *P*_RT_ = 0.02, uncorrected) (see Figure 1). Furthermore, the increased theta power in the bilateral central and temporal regions (*R*_LC_ = −0.358, *P*_LC_ = 0.014, *R*_RC_ = −0.306, *P*_RC_ = 0.036, *R*_LT_ = −0.337, *P*_LT_ = 0.021, *R*_RT_ = −0.312, *P*_RT_ = 0.033, *FDR* corrected) was significantly correlated with worse HVLT-DR scores (see Figure 2).

There were no significant differences in demographic information, delayed recall performance, or theta power between ApoE ε4 carriers and non-carriers in either the MCI group or control group (see Supplementary Table 1).

**FIGURE 3:**
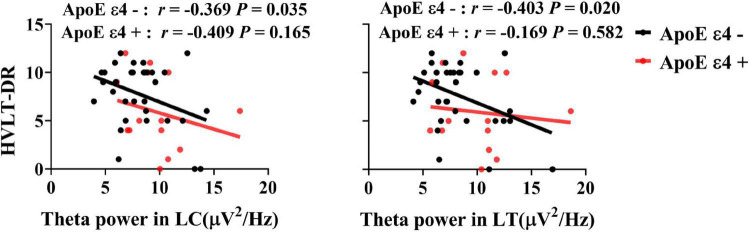
Associations between the delayed recall performance and the theta power in the ApoE ε4 non-carriers and carriers. There was a significant correlation between HVLT-DR scores and theta power in the left central and temporal regions in ApoE ε4 non-carriers (*R*_LC_ = −0.369, *P*_LC_ = 0.035, *R*_LT_ = −0.404, *P*_LT_ = 0.02, uncorrected), but not in ApoE ε4 carriers. HVLT-DR, Hopkins Verbal Learning Test-Delayed Recall; LC, left central area; LT, left temporal lobe.

Significant associations between HVLT-DR scores and theta power in the left central and temporal areas were observed in ApoE ε4 non-carriers (*R*_LC_ = −0.369, *P*_LC_ = 0.035, *R*_LT_ = −0.404, *P*_LT_ = 0.02, uncorrected) but not in the carriers (all *P*s > 0.05) (see Figure 3).

## Discussion

The present study investigated the associations of EEG theta rhythm with delayed recall and the effects of ApoE genotype on these associations in elderly individuals without dementia. The results showed that there was increased theta power and impaired delayed recall in the MCI patients. Consistent with our hypothesis, worse delayed recall performance was associated with higher theta power in the bilateral central and temporal areas, especially in ApoE ε4 non-carriers and not in carriers.

First, as expected, we verified the increased theta power in MCI patients, and found that theta power in the bilateral central and temporal area was negatively correlated with delayed recall performance, which were consistent with previous evidence (Jacini et al., 2018; Hughes et al., 2019). The theta rhythm has been well accepted to be linked with memory integration, from the encoding of new information to the retrieval of stored items (Kirk and Mackay, 2003; Lisman, 2005). The central and temporal areas play a central role within the memory circuitry. Theta power in the MTL increased when new memories were included in an existing mnemonic representation (Backus et al., 2016). In this regard, the association between theta rhythm and delayed recall is reasonable.

Similarly, an increased relative theta band power more intensely over the bilateral middle temporal gyri has been described in a magnetoencephalography (MEG) study in patients with MCI. Jacini et al. also found significant differences in functional connectivity in the theta bands in both temporal poles in patients with MCI (Jacini et al., 2018). Higher EEG theta amplitude in parietal, occipital, temporal and limbic areas has been found in AD (Babiloni et al., 2006), as well as in early AD in almost all cortical regions, including the hippocampi (Engels et al., 2016). Such evidence shows that the theta band yields information that is specifically relevant for MCI and AD.

Taking the ApoE genotype into consideration, there were no differences in delayed recall performance or theta power between the ApoE ε4 carriers and non-carriers within the two groups. The findings are partially in agreement with previous evidence. For example, some previous studies reported there were no significant difference on delayed recall in the AD patients who were ε4 allele carriers and non-carriers (Wang J. et al., 2015; Wang X. et al., 2015). Two recent studies reported that the detrimental effects of ApoE ε4 on delayed recall were modulated by the hippocampal volume/intracranial volume ratio (HpVR) (Wang et al., 2019), and the heterogeneous impact of ApoE ε4 on verbal memory performance was modulated by MTL resting-state cerebral blood flow and entorhinal cortical thickness (Hays et al., 2019). Gutiérrez-de Pablo et al. (2020) found no differences in EEG complexity between AD ApoE ε4 carriers and non-carriers, but brain activity in HCs who were ApoE ε4 carriers was more complex than that in non-carriers. Another report showed that both ApoE ε4 carriers and non-carriers in the normal control group had normal EEG, and no significant differences in quantitative EEG (QEEG) data were found between ApoE ε4 carriers and non-carriers (Jiang et al., 2011). Patterson et al. (2020) found no significant difference in theta power between carriers and non-carriers among patients with MCI, but ApoE ε4 carriers had reduced theta/gamma coupling during a working memory task. Therefore, we speculate that there should be difference in some other cognitive domains and EEG oscillations dynamics affected by the genetic factors. The absence of ApoE ε4’s effect on delayed recall or theta power in our results may also be reasonable, except that the sample size was small.

Interestingly, the statistically significant associations between theta power in the left central, temporal arears and delayed recall performance in this elderly population was found only in ApoE ε4 non-carriers and not in carriers. The lateralization of association with delayed recall might support the notion that the left hemisphere showed accentuated alterations in early stages before AD development. Spectral analysis of the EEG showed that alterations in the left temporal regions in early stages of the disease might be more important (Smailovic et al., 2018), since left hemisphere metabolic activity appears to be affected earlier than right hemisphere metabolic activity (Braak and Braak, 1997). This findings only in the non-carriers was in line with a previous study, to some degree, in that they found that the strengths of the posterior cingulate cortex connections with the right precuneus, insula, and fusiform area were positively correlated with MMSE scores in ApoE ε4 non-carriers but not in ApoE ε4 carriers (Zhu et al., 2019). Based on previous evidence, the ApoE ε4 allele genotype leads to distinct brain functional temporospatial alterations. For instance, ApoE ε4 carriers showed the opposite trajectory in default mode network (DMN) connectivity across the AD spectrum compared to ApoE ε4 non-carriers (Zhu et al., 2019). In another report, ApoE ε4 carriers among cognitively unimpaired subjects exhibited a lower network radius and modularity in the whole-brain DMN (Kuang et al., 2020). Although we did not find differences in theta power between carriers and non-carriers in the current study, it might be reasonable to speculate that delayed recall performance in carriers is correlated with other neural substrates modulated by the ApoE ε4 allele genotype.

However, several issues need to be further addressed. First, the sample size was relatively small in this study, although the current sample size met the minimum requirement on the correlational analyses of the neurophysiological indexes as reported previously (Vozzi et al., 2021). Future work expanding sample size are warranted to verify the current findings. Also, the group sizes of carriers and non-carriers was not balanced in this study, which limits efforts in the exploration of dose-related effects. An imbalance in sample size has been observed in numerous genetic neuroimaging studies (Sheline et al., 2010; Aad et al., 2015; Chiesa et al., 2019). Additional studies are warranted in the future to determine the exact relationships among episodic retrieval performance, dynamics in brain oscillations and ApoE ε4 gene dose by recruiting a large cohort of participants. Second, some findings with statistical significance failed to be corrected by the multiple comparisons, which may increase the false positive risks, conclusions should be interpreted with caution. Third, we only focused on theta power as the EEG variables of interest in the current pilot study. Given that the roles of different frequency oscillations and cross-frequency interactions in the integration of cognition, other EEG variables and features would probably be affected by ApoE ε4 status. Thus, more EEG dynamic characteristics which get involved in the mechanisms of episodic retrieval in both ApoE ε4 carriers and non-carriers should be explored in our next work. Fourth, considering the relatively low spatial resolution of EEG, source-constructed methods and combination with MRI would help reveal the neural localization more precise in future.

## Conclusion

Taken together, in the present study, we verified the increased theta power in MCI patients, and the negative relationships between theta power in the bilateral central and temporal area and delayed recall performance in the elderly individuals without dementia. Interestingly, we noticed that worse delayed recall performance was associated with higher theta power in the left central and temporal areas, especially in ApoE ε4 non-carriers and not in carriers. These current initial observations might provide new evidence for understanding disturbances in brain oscillations and cognitive decline. In combination with the ApoE ε4 genotype results, our findings on distinct neural correlates of memory decline between carriers and non-carriers provide new potential targets for the exploration of biomarkers and neuromodulation of cognitive impairment in the future.

## Data Availability Statement

The original contributions presented in the study are included in the article/Supplementary Material, further inquiries can be directed to the corresponding author.

## Ethics Statement

The studies involving human participants were reviewed and approved by the Peking University Sixth Hospital. The patients/participants provided their written informed consent to participate in this study.

## Author Contributions

JW and TS contributed equally. JW contributed to the study design, analysis and interpretation, drafted, and revised the manuscript. TS contributed to the data collection, analysis, and drafted the manuscript. YZ contributed to the data collection. XY and HW conceived the study and contributed to critical revisions of the manuscript. HW had primary responsibility for the final content. All authors read and approved the final manuscript.

## Conflict of Interest

The authors declare that the research was conducted in the absence of any commercial or financial relationships that could be construed as a potential conflict of interest.

## Publisher’s Note

All claims expressed in this article are solely those of the authors and do not necessarily represent those of their affiliated organizations, or those of the publisher, the editors and the reviewers. Any product that may be evaluated in this article, or claim that may be made by its manufacturer, is not guaranteed or endorsed by the publisher.

## Footnotes

1
http://sccn.ucsd.edu/eeglab/
