# Supplementary material for: Distinct Effects of the Apolipoprotein E ε4 Genotype on Associations Between Delayed Recall Performance and Resting-State Electroencephalography Theta Power in Elderly People Without Dementia
Source: Front Aging Neurosci. 2022 May 26;14:830149. doi: 10.3389/fnagi.2022.830149 (PMC9178171; doi:10.3389/fnagi.2022.830149)
Supplement: Supplementary file 1 [file Table_1.DOCX]

Table S1. Demographic, cognitive performance and theta power in the subgroups of MCI and controls, by ApoE ε4 status

Table S1. Demographic, cognitive performance and theta power in the subgroups of MCI and controls, by ApoE ε4 status.

|  | MCI | | | HC | | |
| --- | --- | --- | --- | --- | --- | --- |
|  | ApoE ε4 carriers  (N=9) | ApoE ε4 non-carriers  (N=14) | *P* | ApoE ε4 carriers  (N=5) | ApoE ε4 non-carriers  (N=19) | *P* |
| age | 70.89±8.536 | 73.07±11.09 | 0.621 | 63.4±5.128 | 67.26±8.218 | 0.332 |
| sex (male/female) | 4/5 | 5/9 | 0.675 | 3/2 | 11/8 | 0.932 |
| education | 13.78±2.539 | 13.43±2.738 | 0.762 | 14±2.236 | 13±2.981 | 0.494 |
| MMSE | 25.78±1.787 | 27.07±1.9 | 0.118 | 29.2±0.837 | 29.37±1.212 | 0.774 |
| MoCA | 22.78±2.635 | 22.5±1.829 | 0.768 | 27.4±0.894 | 27.79±1.228 | 0.516 |
| HVLT-DR | 4.44±3.539 | 6.14±3.820 | 0.297 | 8.60±3.050 | 8.74±2.353 | 0.914 |
| Theta power |  |  |  |  |  |  |
| Left frontal lobe | 9.52±1.703 | 9.40±3.525 | 0.7768 | 9.78±4.428 | 7.76±1.917 | 0.3555 |
| Right frontal lobe | 9.05±1.548 | 9.40±3.693 | 0.8255 | 9.53±4.558 | 7.56±2.162 | 0.4343 |
| Left central area | 9.68±1.641 | 9.48±3.336 | 0.8749 | 9.52±4.569 | 7.64±2.007 | 0.5223 |
| Right central area | 9.42±1.869 | 9.37±3.379 | 0.729 | 9.25±4.408 | 7.39±1.975 | 0.5223 |
| Left temporal lobe | 10.22±2.379 | 9.38±3.781 | 0.729 | 9.48±5.217 | 7.49±1.821 | 0.5696 |
| Right temporal lobe | 9.20±1.793 | 9.58±3.630 | 0.9749 | 9.30±5.234 | 7.58±1.871 | 0.887 |
| Left parieto-occipital lobe | 9.35±2.208 | 9.71±4.108 | 0.9749 | 9.60±4.977 | 7.29±2.277 | 0.3937 |
| Right parieto-occipital lobe | 8.99±2.147 | 9.72±4.103 | 0.6366 | 8.68±4.683 | 7.18±2.395 | 1 |

Note: MMSE: Mini-Mental State Examination; MoCA: Montreal Cognitive Assessment; HVLT-DR: Hopkins Verbal Learning Test-Delayed Recall.
